# Smart Coatings Prepared via MAPLE Deposition of Polymer Nanocapsules for Light-Induced Release

**DOI:** 10.3390/molecules26092736

**Published:** 2021-05-06

**Authors:** Valentina Marturano, Francesco Abate, Veronica Ambrogi, Valeria Califano, Pierfrancesco Cerruti, Giovanni Piero Pepe, Luciano R. M. Vicari, Giovanni Ausanio

**Affiliations:** 1 Institute for Polymers, Composites and Biomaterials, National Research Council of Italy (IPCB-CNR), Via Previati 1/E, 23900 Lecco, Italy; valentina.marturano@ipcb.cnr.it (V.M.); cerruti@ipcb.cnr.it (P.C.); 2 Department of Chemical, Materials and Production Engineering, University of Naples Federico II, P. le Tecchio 80, 80125 Naples, Italy; francesco.aba@libero.it; 3 Istituto Motori CNR, Via G. Marconi 8, 80125 Naples, Italy; valeria.califano@cnr.it; 4 CNR-SPIN and Department of Physics “E. Pancini”, University of Naples “Federico II”, P. le Tecchio 80, 80125 Naples, Italy; gpepe@na.infn.it (G.P.P.); vicari@unina.it (L.R.M.V.); ausanio@unina.it (G.A.)

**Keywords:** light-responsive nanocapsules, microneedles, smart coatings, MAPLE, controlled release

## Abstract

Herein, smart coatings based on photo-responsive polymer nanocapsules (NC) and deposited by laser evaporation are presented. These systems combine remotely controllable release and high encapsulation efficiency of nanoparticles with the easy handling and safety of macroscopic substrates. In particular, azobenzene-based NC loaded with active molecules (thyme oil and coumarin 6) were deposited through Matrix-Assisted Pulsed Laser Evaporation (MAPLE) on flat inorganic (KBr) and organic (polyethylene, PE) and 3D (acrylate-based micro-needle array) substrates. SEM analyses highlighted the versatility and performance of MAPLE in the fabrication of the designed smart coatings. DLS analyses, performed on both MAPLE- and drop casting-deposited NC, demonstrated the remarkable adhesion achieved with MAPLE. Finally, thyme oil and coumarin 6 release experiments further demonstrated that MAPLE is a promising technique for the realization of photo-responsive coatings on various substrates.

## 1. Introduction

Coatings, as the primary barrier between a substrate and the external environment, are the ultimate tool for materials protection against bacteria [1], fouling [2], UV light [3], and corrosive substances [4]. Technological progress is thriving towards the design of active coatings, which are based on stimuli-responsive materials and are able to “respond” to external stimuli through a modification of their properties, including shape, morphology, chemical reactivity, etc. [5,6]. Triggered release is one of the most exploited routes for those applications that require an active agent to be available only under specific conditions [7,8]. In this scenario, biomedical and pharmaceutical industries are in constant search of advanced systems for on-demand release of active agents, to design smart appliances such as bandages [9], prostheses [10], and other medical devices [11].

Recently, we have been focusing on the UV-responsive release of natural actives from polymer nanocapsules (NC) based on azobenzene-containing polymers and we have reported the exploitation of such systems in both liquid phase [12] and as a coating layer on polymeric substrates [13]. This UV-responsive system was later successfully implemented by applying a chemical modification to the azobenzene moiety in order to obtain visible-light- responsiveness [14]. The active substance employed in the aforementioned systems is thyme essential oil (TEO), extracted from *Thymus vulgaris*, a well-known compound with confirmed antimicrobial [15], antioxidant [16], and anti-inflammatory [17] properties. The application of the so-prepared nanocapsule-based coatings as delivery platform of bioactive compounds has several drawbacks [18]. In particular, three main critical points can be identified in the nanocapsules’ purification, dispersion and deposition steps. First, the thyme oil-loaded capsules were obtained via polycondensation in miniemulsion using Mowiol 18-88, a polyvinyl alcohol with a rather high mean molecular weight (c.a. 130,000 Da), as primary surfactant, and their purification represented a challenging procedure. Moreover, the purified NC were more difficult to disperse in liquid media, and finally the coating process, performed by drop casting, often results in poor quality and defects in the final coated layer. For this reason, a more reproducible and reliable deposition technique is needed to gain better control of the ultimate properties of the coating layer. The Matrix-Assisted Pulsed Laser Evaporation (MAPLE) technique was developed in late 1990s at the U.S. Naval Research Laboratory [19] to provide a gentler and less damaging method than its precursor Pulsed Laser Deposition (PLD) for transferring a delicate condensed phase (i.e., a polymeric or organic target) into vapor phase and then collecting it onto a substrate in the form of a thin or ultrathin film. The basic apparatus is similar to that used for PLD, with two substantial differences [20,21]: the target material is diluted (<5 wt.%) in a volatile solvent and is frozen before irradiation. The substrate is placed in front of the target inside a high-vacuum chamber.

In MAPLE, organic and polymer molecules are surrounded by a high number of solvent molecules of the solid matrix, which absorb the laser photons in their place upon irradiation. By photothermal and photochemical effects, the frozen matrix fractures away in clusters with solute molecules entrapped in it. During clusters’ run from target to substrate, volatile solvent molecules are evacuated by the vacuum pump, and a pure solute film forms on the collection surface. This mechanism has been predicted by molecular dynamics simulations, which indicated that ejection of single molecules from the target does not occur [22]. It could also explain the observed formation of droplets in deposited films when part of the solvent molecules is evacuated only after impinging onto the substrate. The droplet formation mechanism has been studied in detail, although a definitive theory has not yet been established. As of now, phase explosion is believed to represent the most significant contribution with respect to other thermally induced ablation mechanisms, namely, normal vaporization at the surface and normal boiling [21]. The major achievement made by the MAPLE technique is exactly the one it was made for: depositing organic and polymeric thin films through laser excitation, preserving functionalization and molecular weights [20]. A wide range of target materials, from simple polymers and polymer blends [23,24] to nanoparticles [25] and more sensitive biomaterials like proteins or carbohydrates, have been successfully deposited [26,27,28,29]. As opposed to solvation methods, a laser deposition method provides some clear advantages. First of all, it is a non-contact, contamination-free process. No direct forces are applied to the substrate, apart from the small ones due to droplet impact. Secondly, it is a pseudo-dry technique: the solvent phase is expected to be pumped away before arriving onto the substrate or at least immediately after. Solvent-wetting and evaporation steps are responsible for non-uniform coatings when drop casting, spray coating, or dip coating are used. MAPLE-deposited films, instead, present an extremely high degree of homogeneity both in thickness and in distribution [20]. Finally, a high control over surface thickness and roughness is obtained, with Root-Mean-Square (RMS) values around 1–100 nm for thicknesses, ranging from tens of nm to some µm depending on the deposition parameters [21]. Residual roughness is mostly due to droplet mechanism effects, but compared to surface finish of solvation methods, the quality gain is remarkable.

This work reports for the first time the use of MAPLE to develop active coatings based on polymer nanocapsules containing essential oils. In particular, the feasibility of MAPLE as a deposition technique for NC loaded with bioactive TEO was tested. The target consisted of a non-purified polyamide NC suspension. This allowed avoiding time-consuming, often inefficient purification procedures and the consequent difficulty of dispersion of the purified NC. The laser wavelength chosen, 1064 nm, was selected to avoid photo-activation of the NC during the deposition, as would possibly happen with UV or visible radiation, so as to keep TEO inside the NC film after the deposition. Inorganic and organic deposition substrates were selected, including potassium bromide (KBr), polyethylene (PE), and acrylate micro-needles, and the coated surface morphology as well as TEO release were studied.

## 2. Results and Discussion

**Figure 1:**
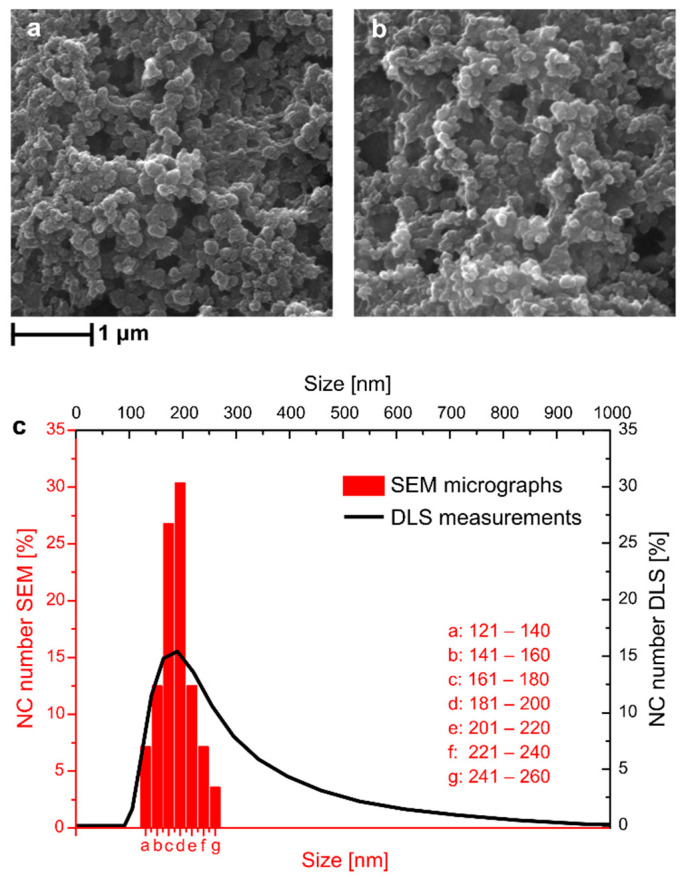
SEM micrographs of NC-TEO before (**a**) and after (**b**) treatment with liquid nitrogen and comparison between DLS and ImageJ analyses of SEM micrographs (**c**).

Photo-responsive core−shell polymer NCs containing both C6 and TEO as core materials, named NC-TEO, were prepared through interfacial miniemulsion polycondensation [12]. The encapsulation efficiency of thyme oil in the NCs was determined using UV−vis spectroscopy, which yielded a value of 84.6 ± 4.3%. The resistance of NC-TEO to low temperatures was tested using liquid nitrogen to quickly freeze them and comparing the appearance of the so-treated NC with the untreated ones. This test was performed to evaluate the ability of NC-TEO to withstand the fast cooling and prolonged freezing that they underwent during MAPLE deposition. As shown in Figure 1, the pristine NC-TEO (Figure 1a) were able to maintain their shape and size unchanged even after three freezing cycles (Figure 1b). In Figure 1c, dynamic light scattering (DLS) measurements are compared with the data gathered from dimensional analysis of SEM micrographs on a sample area containing about 100 NC. The results show that SEM and DLS size curves have different distributions, as the DLS curve is more extended towards higher size values. This can be due to aggregation phenomena occurring when capsules with low surface charge were dispersed in a liquid medium. The DLS equipment detected the capsule agglomerates as bigger particles with higher hydrodynamic radius. However, both curves show a peak at around 180 nm, confirmed as the mean NC size.

**Figure 2:**
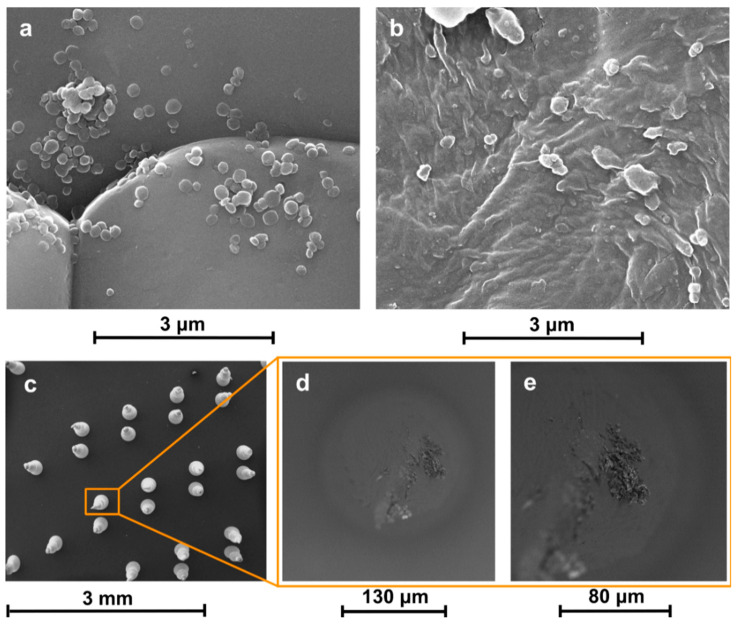
SEM micrographs of NC-TEO deposited via MAPLE on KBr (**a**), PE (**b**), and acrylate microneedles (**c**–**e**).

SEM images of NC-TEO deposited on a KBr disk (Figure 2a) revealed that azobenzene-based NCs were successfully transferred to the substrate without significant alteration in shape. Deposition on PE (Figure 2b) led to the formation of a composite film, with residual PVA surfactant acting as a matrix that gathered and wrapped the capsules, incorporating them in a compact coating.

Agglomerates could be found throughout the sample after deposition with the MAPLE technique. Such features are not rare in MAPLE deposits: complex surface morphologies have often been observed [30,31,32,33]. Actually, laser irradiation causes the explosive disintegration of the MAPLE target, with the ejection of a mixture of vapour-phase molecules, small molecular clusters, and droplets. The solvent evaporated during the target-to-substrate flight, increasing NC concentration in the ejected droplets and clusters, so that micrometric aggregates formed. However, MAPLE dramatically improved the capsules dispersion on the substrate compared to coatings obtained via drop casting (as proved by SEM in Figure 1a). We think that the quality of the coating can still be improved by further diluting the NC solution, so that each ejected droplet contains a smaller number of NC, which would strongly reduce the size of the agglomerates.

NC-TEO capsules were also deposited on a microneedle array, as shown in Figure 2c. Images taken along the edge of a single needle with varying focal distance (Figure 2d,e) proved that the target material reached the whole substrate surface notwithstanding its inclination.

**Figure 3:**
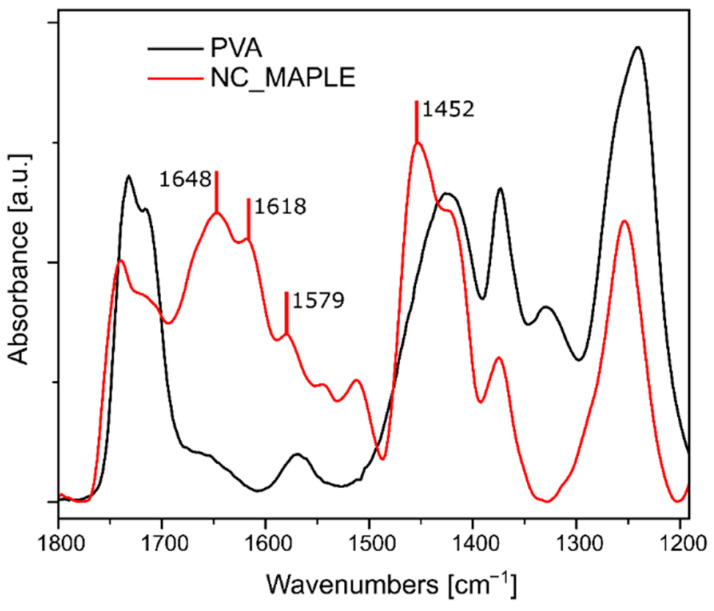
FTIR spectrum of NC deposited via MAPLE compared with the PVA spectrum.

In order to confirm the successful deposition of NC on the substrate, the coated KBr disc was characterized by FTIR spectroscopy. Transmission experiments were hindered by the presence of micrometric clusters in the NC-coated layer inducing scattering phenomena and resulting in a noisy FTIR signal. Therefore, after deposition, the coating was gently scratched off from the KBr disc with a scalpel, and the resulting powder, containing the deposited NC, was placed on the FTIR–ATR diamond and analyzed. As shown in Figure 3, the spectrum obtained was compared with the spectrum of PVA Mowiol 18-88 used as a surfactant in the NC preparation. The two spectra present several common peaks around 1730, 1424, and 1240 cm^−1^. In particular, the peak at 1730 cm^−1^ was attributed to C=O stretching of the acetyl groups of PVA [34]. The spectrum of the layer scratched by KBr, moreover, presents a few peaks, which confirm the presence of NC deposited via MAPLE: the peaks at 1648 and 1579 cm^−1^ correspond to the amide I and II characteristic peaks [35], while that at 1452 cm^−1^ was attributed to the N=N stretching mode in para position on the azobenzene ring [36].

**Figure 4:**
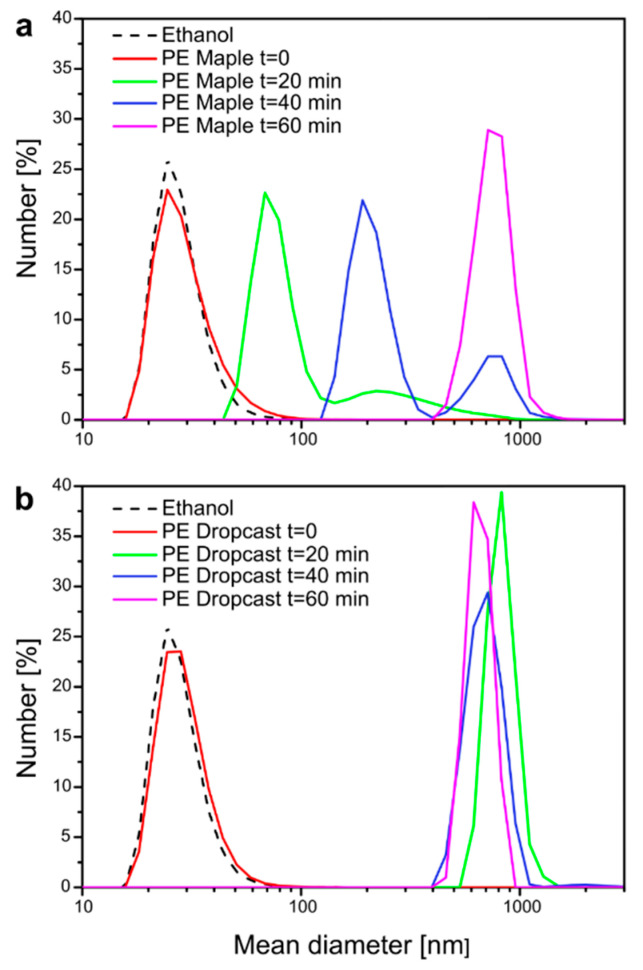
DLS analysis of NC deposited via MAPLE (**a**) compared with NC deposited via drop casting (**b**).

The main advantage of MAPLE deposition is the high kinetic energy employed, which allows a soft material to be impressed on a substrate without being stressed or damaged. Compared to other methods involving liquid evaporation, MAPLE offers a better adhesion and dispersion of the deposited material [20,21]. A comparison between the stability of the NC coating resulting from MAPLE with that obtained via simple drop-casting is reported in Figure 4. Two PE films, coated by MAPLE and drop casting, respectively, were placed in a glass cuvette filled with an 80% EtOH solution and kept in darkness for 30 days. It is worth pointing out that the solution containing the MAPLE-coated film was the same used as unirradiated sample in the release experiments presented below. The two solutions were then periodically analyzed by DLS to monitor the presence of NC in the release medium, hence, to assess qualitatively the stability of the coating. To facilitate the removal of the coating layer from the substrate, the cuvettes were placed in an ultrasound bath filled with ice water and analyzed after 20, 40, and 60 min of sonication. In both cases, ultrasound treatment promoted the release of the coatings from the PE films, as shown in Figure 4. At t = 0, a peak in both samples, indicative of the presence of small particles (mean size = 20–40 nm) could be associated with small amounts of impurities in the reference EtOH sample, as confirmed by the analysis of pristine EtOH (dashed line in Figure 4 top and bottom). However, the two coatings responded differently to the combined treatment consisting in 80% EtOH soaking and sonication. The MAPLE coating appeared to be fairly resistant to the harsh solvo-mechanical stress (Figure 4a): at first (t = 20 min), the treatment was able to pull away from the PE substrate 70–80 nm-sized debris, likely consisting of smaller NCs or PVA fragments loosely bound to the surface. At t = 40 min, DLS detected the presence of 200 nm particles; this characteristic dimension is very similar to the one attributed to NC-TEO, probably indicating that at this time, the first NC were being strayed from the PE film. At t = 60 min, sub-micrometer particles (around 700–800 nm) were detected, hinting at the presence of the wrecking coating dispersed in the EtOH solution. The same large debris appeared in the drop-casted coating after only 20 min of sonication (Figure 4b), suggesting its faster mechanical failure due to poor cohesion of the coating. This result was ensured by MAPLE ability to promote strong cohesion between the NC and the substrate, a key factor for the production of mechanically stable coatings.

**Figure 5:**
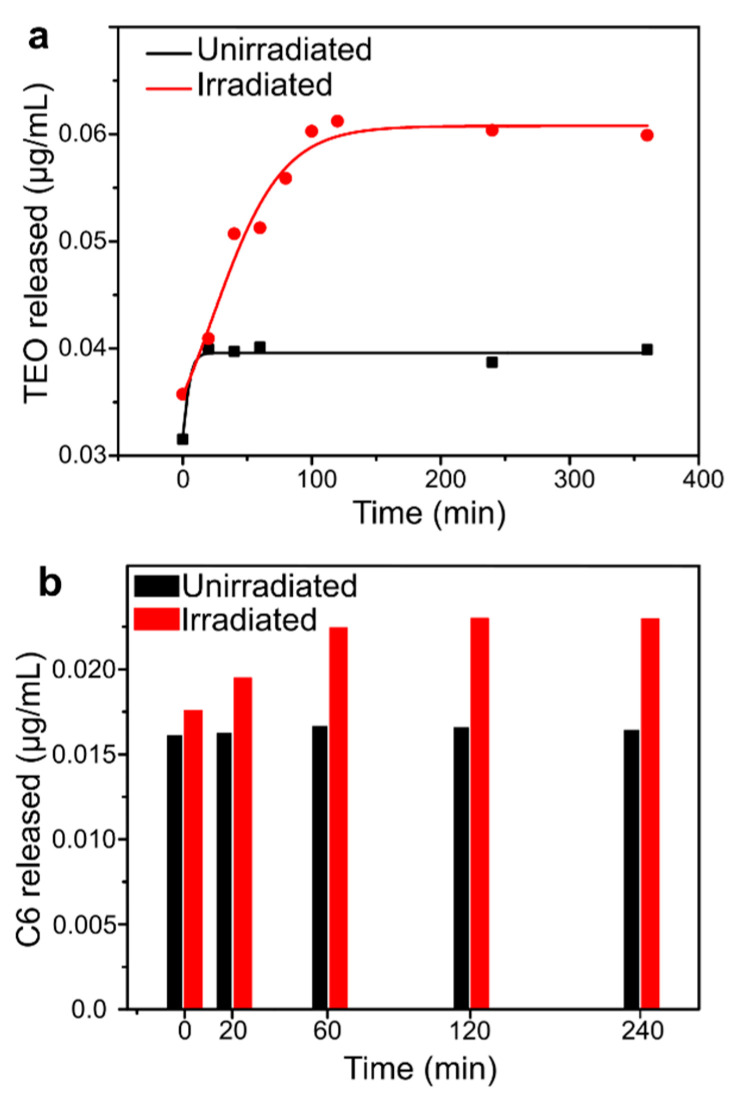
Release kinetics of TEO (**a**) and C6 (**b**) from MAPLE-deposited NC.

Previous studies confirmed that, upon UV light irradiation, capsules can release their cargo materials as a consequence of a squeezing effect produced in the polymer shell by azobenzene trans-cis photo-isomerization [37]. The UV light-triggered release kinetics of C6 and TEO from NC-TEO deposited on PE was studied by UV–vis spectroscopy and spectrofluorimetry, and the results are reported in Figure 5a. The solid lines reported in Figure 5a represent a guide to the eye to fit the data obtained via UV–vis spectroscopy. The release curve of UV-irradiated sample follows a two-step trend, with a high release rate during first 40 min, a plateau between 40 and 60 min, and a slower release rate from then on. After 100 min, the releasing capacity of NCs reached its limit, thus the concentration of TEO in the release medium stopped increasing. Rather, a smaller quantity of TEO leached from the unirradiated sample during the first 20 min, then the increase in TEO concentration immediately stopped. Comparing the two release curves, it is clear that the capsules retained their functionality as stimuli-responsive carriers after being deposited via MAPLE. This result is not trivial, since the irradiation of the target with the high-power laser beam could cause the breakage of the NCs and the release of their content during MAPLE deposition. The possible reason is in the choice of the laser wavelength, 1064 nm, which was unable to cause photoactivated release of the polymer capsules. However, the effect of different laser wavelengths on the release of MAPLE-manufactured coatings should be further studied.

In Figure 5b, coumarin-6 (C6) release is reported as a function of irradiation time. As we can see, at t = 0, a significant amount of C6 (0.0176 and 0.0161 µg/mL for irradiated and unirradiated samples, respectively) was already present in the release media. A small increase in the C6 concentration was detected upon irradiation with UV light, while the unirradiated sample maintained approximately a constant concentration of C6 in the solute.

It appears from the above discussion that the morphological, chemical, and photo-responsive properties of NC-TEOs persisted even when after deposition in film shape. Given the complexity of the above characteristics, it was not at all obvious that MAPLE laser deposition, although a gentle technique, would preserve the photoactivation features related to the chemical properties. Preserving all three characteristics is therefore essential. This was possible because in the present study, after an initial evaluation to identify the laser pulse energy sufficient to induce ablation of the target, deposition conditions such as energy, pulse frequency, and wavelength were chosen to preserve all the properties of the NCs. Higher pulse powers, in fact, led to the destruction of the physico-chemical properties of the NCs, a finding not reported here. In the future, the tailoring of coating uniformity can be studied, for example, by tweaking the target–substrate distance, deposition time, residual pressure, and substrate temperature.

## 3. Materials and Methods

### 3.1. Materials

1,8-diaminoctane (DAO), 1,3,5-benzenetricarbonyl, trichloride (BTC), sodium hydrogenocarbonate, Mowiol, 18-88 (M18-88, MW = 130 kDa), and coumarin-6 (C6) were purchased from Sigma-Aldrich (Milan, Italy) and used without further purification. 4,4′-bis(chlorocarbonyl)azobenzene (CAB) was synthesized according to a previously reported procedure [38]. TEO was purchased from Farmalabor, (Canosa di Puglia, Italy), used without further purification, and coded as NCT. KBr was purchased from Sigma Aldrich, corona-treated linear low-density polyethylene (PE) film was prepared according to a previously reported procedure [13], the acrylate film-supported array of micrometric needles was kindly donated by ST-Microelectronics [39,40].

### 3.2. Preparation and Characterization of TEO Capsules before Deposition

TEO-loaded nanocapsules (NC-TEO) were obtained by interfacial polycondensation in an O/W miniemulsion. Two solutions were separately prepared, an aqueous and an organic phase. An aqueous phase (mother solution) was prepared by dissolving 1 wt% of the surfactant Mowiol 18–88 in 100 mL of milliQ water. From the mother solution, two different solutions were obtained: Solution 1 consisted of 30 mL of mother solution, Solution 3 was prepared by dissolving 0.061 g of DAO and 0.071 g of the acid quencher NaHCO_3_ in 6 mL of mother solution. The organic phase (Solution 2) was prepared by dissolving 0.125 g of CAB, 3.35 mg of BTC, and 4.0 mg of C6 in 6 mL of TEO (1:6, *v*/*v* o/w ratio). To dissolve azobenzene in TEO, the solution was kept under magnetic stirring (500 rpm) until the solid dissolved, and the red solution appeared clear.

To obtain the O/W emulsion, Solution 2 was added dropwise to Solution 1 under magnetic stirring (1000 rpm) and kept under stirring for 30 min. Solution 3 was then slowly added dropwise to the emulsion formed by solutions 1 and 2. Stirring was then set at 500 rpm and kept overnight.

TEO shows a well-recognizable absorbance peak at around 278 nm, and its encapsulation efficiency (EE) was assessed via UV−vis spectroscopy using a set up double-crossing protocol perfected in a previous work [12]. An indirect value of encapsulation efficiency (EEi) was measured as the difference between the amount of TEO that was used in capsule preparation and the quantity of free oil detected in the supernatant after filtration. Moreover, direct encapsulation efficiency was also assessed by measuring the amount of EO in the supernatant after extraction with DMSO at high temperature using EEi as a corrective factor.

NC-TEO average size was determined through Dynamic Light Scattering (DLS) analysis using a Zetasizer Nano ZS (Malvern Instruments, Malvern, UK). The analysis was performed at 25 °C at a scattering angle of 173°. Scanning Electron Microscopy (SEM) was carried out using a FEI Quanta 200 FEG instrument in high-vacuum mode, equipped with a Large Field Detector (LFD) operating with acceleration voltage ranging between 15 and 20 kV. A few milliliters of NC-TEO solution were cooled with liquid nitrogen for three times to mimic the thermal stress that occurs in the MAPLE setup and observe any changes in NC morphology or dimension. Pristine and liquid nitrogen-treated samples were deposited via drop casting on two SEM aluminum stabs and metallized with an AuPd coating.

### 3.3. MAPLE Deposition

**Figure 6:**
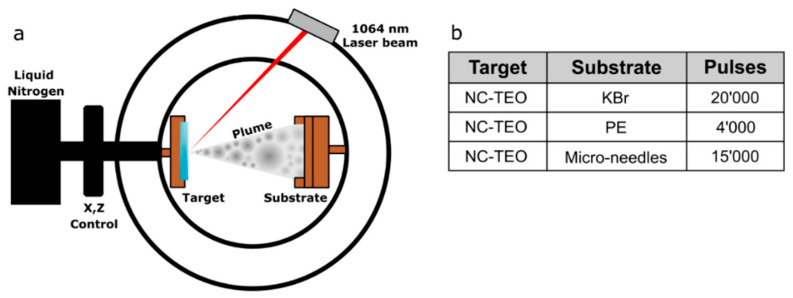
Schematic of MAPLE equipment (**a**) and deposition parameters for the three selected substrates (**b**).

A MAPLE set-up equipped with a Nd:YAG 1064 nm laser and two pumps (a rotary pump for bootstrap and a turbomolecular pump for high vacuum) was used. A schematic of the MAPLE apparatus is presented in Figure 6a.

The target holder, a copper vessel of 2 mL capacity, was filled with NC-TEO solution and placed in thermal contact with liquid nitrogen. Once the target was frozen, the vacuum chamber was evacuated. The Nd:YAG pulsed laser was operated at 1064 nm. The laser beam reached the target at 45°, partially focused to an ellipsoidal area of about 1.0 mm × 1.4 mm. The target was moved with a 2D translation system in order to avoid overheating or drilling. The laser beam scanned a target area of about 1.5 cm^2^. The following parameters were kept constant for all depositions: pressure inside the chamber ~10^−6^ torr, target temperature ~−133 °C, target–substrate distance 8 mm, pulse repetition rate 4 Hz, pulse duration 7 ns, laser pulse energy 410 mJ/pulse. The laser power was set just above the solution ablation threshold. In the target, the nanocapsules concentration in the aqueous solution was 0.04 g/mL. Further parameters for each deposition are listed in Figure 6b. The rationale for choosing the substrates was as follows: KBr and PE were tested as flat inorganic and organic substrates for MAPLE deposition of NC-TEO. Moreover, micro-needles were tested to verify the quality of the deposition on 3D surfaces.

### 3.4. Characterization of NC-TEO Coating

A FEI Quanta 200 FEG SEM was used for the direct observation of the coated surfaces. MAPLE-deposited NC-TEO samples were directly observed without further modification. Samples were analyzed in high-vacuum mode after Au–Pd sputtering with an Emitech K575. The same procedure was used to prepare NC-coated microneedles, analyzed with a FEI Phenom desktop SEM.

A Spectrum 100 Perkin–Elmer FTIR spectrophotometer equipped with a single bounce diamond ATR device, acquiring 32 scans at 4 cm^−1^ resolution, was used to confirm the composition of the deposited NC layer obtained via MAPLE on KBr. The coating was scratched off with a scalpel, collected, and placed on the ATR diamond to be analyzed. The obtained FTIR spectrum was compared with the ones of pure KBr and Mowiol 18-88, the surfactant used in NC preparation. DLS was employed to compare the stability of the NC coating resulting from MAPLE with the one obtained via simple drop casting. Two PE films, coated by MAPLE and drop casting, respectively, were placed in a glass cuvette filled with 2 mL 80% EtOH solution and kept in darkness for 1 month, purposely the solution used was the same previously employed as unirradiated sample in the release studies reported in the following section. The two solutions were periodically analyzed by DLS to monitor the presence of NC in the release media. To accelerate NC removal from the film surface, the cuvettes were placed in an ultrasound bath filled with ice/water and analyzed with DLS after 20, 40, and 60 min.

To demonstrate the UV light-sensitive behavior of the fabricated NCT coatings, release experiments were carried out as follows. A circular (approximately 1 cm diameter) NCT-coated PE film obtained via MAPLE was carefully cut in half to produce two almost identical samples. Both were placed on the flat bottom of a quartz cuvette, with the coated layer facing down, and were both filled with 2 mL of 80% ethanol solution, used as release medium to promote thyme oil dissolution. To start the release study, the as-prepared samples were analyzed using a JASCO Mod. V570 UV–vis spectrophotometer with a double-beam/single-monochromator optical system. Then, one cuvette was suspended about 3 cm above a UV lamp (λmax = 360 nm) with the bottom facing the light. Both irradiated and unirradiated solutions were analyzed every 20 min for 4 h until the absorbance intensity stopped increasing over irradiation, indicating that release had ceased. Data were elaborated with Origin Pro 9.1 64-bit.

In a similar experiment, the two solutions were then analyzed via spectrofluorimetry using a PerkinElmer LS-55 fluorescence spectrometer (λex = 390 nm, λem = 500 nm). In this case, the release experiment was carried out during 240 min and until the increase in fluorescence intensity plateaued, signifying the end of C6 release.

## 4. Conclusions

Photo-active coatings based on NC-TEO were successfully manufactured on various substrates via MAPLE deposition. The capsules appeared well dispersed and effectively anchored to the substrate surface. Moreover, they did not show any apparent change in their shape and dimensions. These features were obtained thanks to the use of MAPLE, which is characterized by the absence of solvent evaporation that avoids particle agglomeration upon drying. The possibility to deposit nano-encapsulated systems via MAPLE on different materials, such as KBr, polyethylene, acrylate microneedles, was demonstrated. The feasibility of this approach was proven for azobenzene-based light-responsive nanocapsules containing TEO, prepared via interfacial polycondensation. It was shown that MAPLE allows the deposition of encapsulated volatile active agents, such as TEO, also preventing their evaporation during coating preparation. Moreover, this technique is suitable even for substrates of different nature and with complex surface morphologies. Indeed, successful transfer of azobenzene nanocapsules to different substrates, including the inclined surface of micro-needles, was achieved. Finally, the results inferred from the release studies upon light irradiation confirmed the successful fabrication of photo-responsive coatings through a facile technique such as MAPLE. Further studies are required to correlate the effect of MAPLE processing parameters (i.e., laser wavelength, pulse conditions) with the efficiency of NC deposition.

## Data Availability

The data presented are available in the manuscript.
